# Exploring the epidemiological impact of attractive targeted sugar bait against malaria in combination with standard malaria control

**DOI:** 10.1016/j.crpvbd.2025.100247

**Published:** 2025-01-29

**Authors:** Nima R. Moghaddas, Mohamed M. Traore, Gunter C. Müller, Joseph Wagman, Javan Chanda, Julian Entwistle, Christen M. Fornadel, Thomas S. Churcher

**Affiliations:** a Medical Research Council Centre for Global Infectious Disease Analysis, Department of Infectious Disease Epidemiology, Faculty of Medicine, Imperial College, London, UK; b Malaria Research and Training Centre, Faculty of Medicine, Pharmacy and Odonto-Stomatology, University of Sciences, Techniques and Technology of Bamako, Bamako, Mali; c Malaria and Neglected Tropical Diseases Program, Center for Malaria Control and Elimination, PATH, Washington, DC, USA; d PATH Malaria Control and Elimination Partnership in Africa, Lusaka, Zambia; e Innovative Vector Control Consortium, Liverpool, UK

**Keywords:** ATSB, Sugar feeding, Vector control, Malaria, Mathematical modelling, Bait

## Abstract

Attractive targeted sugar bait (ATSB) is a potential new vector control tool that exploits the sugar-feeding behaviour of mosquitoes. Little is known about the factors which drive ATSB efficacy, either as a standalone vector control tool or in combination with existing intervention strategies. It has been suggested that the percentage of wild mosquitoes caught fed on dye-containing sugar baits without the toxin could provide an entomological correlate of the potential epidemiological benefit of ATSB. A transmission dynamics mathematical model is combined with data from wild mosquitoes to investigate the relationship between the mosquito dyed fraction, bait-feeding rate and the potential epidemiological impact of ATSB in the presence of standard malaria control. The dyed fraction in Mali varies substantially in space and time (mean 0.34, standard deviation 0.15), causing estimates of the bait-feeding rate to be highly uncertain, especially in areas with existing vector control tools. The model indicates the dyed fractions observed in field experiments were broadly predictive of the reductions in mosquitoes caught when ATSB stations were deployed at scale in Mali (*R*^2^ = 0.90). Model projections suggest that if these bait-feeding rates were observed in all mosquitoes, then the widespread use of ATSB could substantially reduce malaria burden alone or in combinations with standard malaria control, though epidemiological impact is likely to vary substantially in different areas. For example, observing a dyed fraction of 5% would indicate a daily bait-feeding rate of 0.024 (range 0.008–0.049) which is projected to result in 0.13 clinical cases averted per person-year (range 0.051–0.22), a 39% efficacy (range 12–66%) in this particular site. Nevertheless, the uncertainty in the relationship between the observed dyed fraction and the true bait-feeding rate, and the underlying biology of mosquito sugar-feeding means that the epidemiological benefit of this new possible intervention remains unclear.

## Highlights

• Attractive targeted sugar bait (ATSB) kills mosquitoes which sugar-feed on it.
• The dyed fraction of mosquitoes is used as a correlate of ATSB efficacy.
• Dyed fraction depends on vector control use, mosquito dynamics and dye properties.
• Model projected mosquito density reproduced the results of a field trial in Mali.
• The epidemiological efficacy of ATSB depends on mosquito sugar-feeding behaviour.

## Graphical abstract

**Figure:**
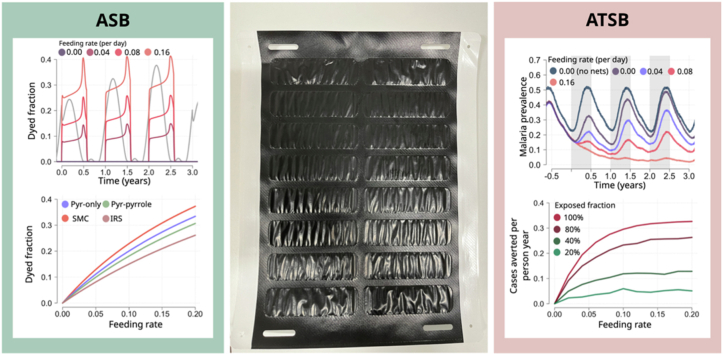

## 1 Introduction

Significant progress has been made in reducing the global burden of malaria over the past two decades, but it remains endemic in sub-Saharan Africa and continues to be a leading cause of morbidity and mortality (WHO, 2023). Statistical models suggest that much of this progress is due to the widespread use of vector control interventions, particularly insecticide-treated nets (ITNs) and indoor residual spraying (IRS) (Bhatt et al., 2015). Several challenges are thought to contribute to the persistence of high malaria burden, including the increasing insecticide resistance in mosquitoes, which reduces the effectiveness of ITNs and IRS (Ranson and Lissenden, 2016). Residual transmission occurs when mosquitoes feed outdoors or during times when people are not directly protected by ITNs and IRS, such as early evening or early morning (Killeen, 2014). This has created a need for complementary tools, like the Attractive Targeted Sugar Bait (ATSB, also referred to as Attractive Toxic Sugar Bait elsewhere), which targets the sugar-feeding behaviour of mosquitoes and can kill mosquitoes in outdoor settings, helping to reduce transmission.

ATSB exploits the sugar-feeding behaviour of mosquitoes by combining a sugar bait with an insecticide (Fiorenzano et al., 2017). They have been used in various forms including as a spray applied over vegetation (Beier et al., 2012; Qualls et al., 2014), in drainage systems (Müller et al., 2010), freshwater cisterns (Müller and Schlein, 2008), or as a bait station which can be attached to the walls of various structures, both indoors (Qualls et al., 2014) and outdoors (Traore et al., 2020). While only female mosquitoes take blood meals, all mosquitoes require sugar to survive (Manda et al., 2007; Barredo and DeGennaro, 2020). Cold anthrone testing for the presence of natural sugar has confirmed that fractions of wild-caught mosquitoes have ingested a sugar meal in the recent past (0.21 for *Anopheles funestus* and 0.10 for *Anopheles gambiae* in Zambia; 0.28 for *An. funestus* and 0.13 for *An. gambiae* in Kenya; and 0.32 for *An. gambiae* in Mali), though this varies by location, sex, blood-fed status, and availability of alternative sugar sources (Traore et al., 2020; Omondi et al., 2022; Chanda et al., 2023). It is hoped that ATSBs can be deployed to such an extent as to substantially increase mosquito mortality and reduce malaria.

Several studies which implemented ATSB against different mosquito species have reported impressive reductions in measures of mosquito abundance (Beier et al., 2012; Stewart et al., 2013; Qualls et al., 2015; Traore et al., 2020). For example, applying ATSB spray over vegetation in uninhabited oases in an arid, malaria-free environment resulted in 97% reductions in populations of *Anopheles sergentii* over 47 days in Israel (Beier et al., 2012). An experimental hut trial of indoor ATSB stations in Tanzania reported 41–48% mortality against *Anopheles arabiensis* compared to 18% mortality in control huts (Stewart et al., 2013). Another study, which placed ATSB stations inside houses in five clusters along the River Niger in Mali, recorded a 90% reduction in female *Anopheles gambiae* (*sensu lato*) over 50 days (Qualls et al., 2015). A larger trial including 14 clusters and extending over two years in Mali, placed ATSBs on the outside of houses and recorded a reduction of 20–57% in female *An. gambiae* (*s.l.*) density, depending on the trap type used (Traore et al., 2020). All this suggests that ATSB can have a significant impact on mosquito populations; however, their impact on malaria epidemiology is currently unknown.

Three phase III cluster randomised control trials (RCTs) to assess the epidemiological impact of ATSBs on malaria in various settings across sub-Saharan Africa have recently been concluded (Attractive Targeted Sugar Bait Phase III Trial Group, 2022). RCTs such as these are expensive, and it is infeasible to repeat them in many countries where malaria is endemic. It is likely that the key entomological metric, the probability that a mosquito feeds on a bait station each day, herein referred to as the bait-feeding rate, will vary substantially according to the relative availability of sugar from bait stations compared to natural sources (Traore et al., 2020; Omondi et al., 2022; Chanda et al., 2023) as well as mosquito population structure and dynamics. There is a need to identify relatively inexpensive correlates of efficacy which could be used by National Malaria Programmes to inform policy decisions on the suitability of ATSB in their regions. For example, the entomological efficacy of ITNs can be evaluated in experimental hut trials. Results of these experimental hut trials have been used to parameterize transmission dynamics models and can reliably predict the results of RCTs evaluating the widespread use of ITNs (Sherrard-Smith et al., 2022). Ideally, these correlates of protection should be assessed alongside RCTs to demonstrate their predictive ability. One potential correlate for ATSB, which offers the possibility for a smaller scale assessment of product efficacy than RCTs, is the dyed fraction of mosquitoes. This is the proportion of mosquitoes that have fed on a modified version of an ATSB (an Attractive Sugar Bait, ASB) which contains a dye instead of a toxin (Traore et al., 2020; Chanda et al., 2023). It is hoped that the dyed fraction in wild-caught mosquitoes will be predictive of the rate mosquitoes feed on bait stations and therefore the entomological efficacy of ATSBs (assuming all mosquitoes that feed on the baits die).

Different approaches have been taken to assess the dyed fraction. In one study, ASB stations were deployed in each village for one night and traps were emptied the following day (Traore et al., 2020). While this approach is hoped to give estimates of dyed fraction that are closer to the daily bait-feeding rate, this can be logistically challenging and difficult to scale, therefore another study deployed ASB stations for an extended period with regular entomological collections to assess the dyed fraction (Chanda et al., 2023). Especially in the latter case, a key distinction should be drawn between the bait-feeding rate and the dyed fraction. The former represents the actual rate of feeding on bait stations and is unknown. The latter can be measured in field experiments and represents the proportion of mosquitoes that were stained and that were caught in a trap. The precise relationship between dyed fraction and bait-feeding rate is unknown and will likely depend on site specific characteristics such as the use of other vector control interventions and the specific mosquito sampling strategy used. For example, if a large proportion of the human population is using ITNs which kill mosquitoes then fewer bait-fed mosquitoes are likely to survive long enough to be caught in a trap, potentially changing the relationship between dyed fraction and the bait-feeding rate.

As a broader malaria control toolkit becomes available it becomes increasingly important to understand how interventions work in combination, given limited budgets. Here, we have developed a modelling framework to estimate the entomological and epidemiological impact of ATSB. The framework initially explores the relationship between the bait-feeding rate and the dyed fraction of mosquitoes caught in ASB studies. This relationship is then used to project the epidemiological impact of ATSB stations from dyed fraction data, with ATSB stations deployed alone or in combination with standard (pyrethroid only) and next generation (pyrethroid-pyrrole) ITNs, IRS, and seasonal malaria chemoprevention (SMC). Finally, we demonstrate how assumptions around mosquito sugar-feeding behaviour and sampling methods could significantly impact the expected impact of ATSB stations.

## 2 Materials and methods

### 2.1 Deterministic ASB model

**Fig. 1:**
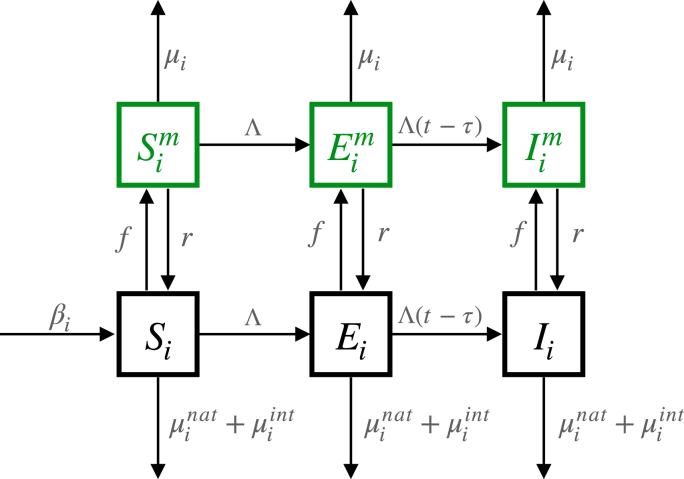
Diagram of the deterministic attractive sugar bait (ASB) feeding model. As adult mosquitoes move through susceptible (*S*), exposed (*E*) and infectious (*I*) compartments they can become marked (denoted by superscript *m*) at any point by feeding on a non-toxic bait station at rate *f*. Parameter $\beta_{i}$ is the number of emerging adult mosquitoes, ${\mu_{i}}^{nat}$ and ${\mu_{i}}^{\mathrm{int}}$ are natural and intervention induced mortality respectively (see main text) and the marking dye decays at rate r. $\mu_{i}$ is the sum of ${\mu_{i}}^{nat}$, ${\mu_{i}}^{\mathrm{int}}$ and ${\mu_{i}}^{dye}$ as described in Equation (2). For the individual-based transmission model $\mu_{i}$ is the sum of ${\mu_{i}}^{nat}$, ${\mu_{i}}^{\mathrm{int}}$ and ${\mu_{i}}^{ATSB}$ as described in Equation (3).

A deterministic compartmental model was developed to determine the relationship between the dyed fraction and the bait-feeding rate in ASB experiments (Fig. 1). This model builds upon a previous framework for anopheline mosquitoes, which was calibrated using mosquito density and rainfall data (White et al., 2011). In essence, larval mosquitoes progress through early instar, late instar, and pupal stages before emerging as adults at a rate determined by the seasonality of local rainfall patterns which vary between sites. Adult mosquitoes initially emerge as susceptible individuals and become exposed to malaria upon biting an infected human. Following a latency period corresponding to the extrinsic incubation period of the parasite, exposed mosquitoes become infectious and can transmit the infection to humans. The model tracks the mean number of susceptible S, exposed E, and infectious I mosquitoes per person. These mosquitoes interact with bait stations, feeding at a rate f and subsequently transitioning to marked susceptible $S^{m}$, marked exposed $E^{m}$, and marked infectious $I^{m}$ states, respectively. The dye ingested during bait station feeding decays at a rate r. Mosquitoes experience constant daily natural mortality at a rate $\mu^{nat}$, which may be augmented by other vector control measures, such as ITNs, contingent upon factors such as net type, net use, net age, and the level of pyrethroid resistance in the mosquito population. The system of linked differential equations can be written as follows,

$$\dot{S_{i}}=\beta_{i}+rS_{i}^{m}-\Lambda_{i}S_{i}-\mu_{i}S_{i}-fS_{i}\text{,}$$

$$\dot{E_{i}}=rE_{i}^{m}+\Lambda_{i}S_{i}-\Lambda_{i}\left(t-\tau \right)S_{i}\left(t-\tau \right)-\mu_{i}E_{i}-fE_{i}\text{,}$$

(1) $${\dot{I}}_{i}=r{I_{i}}^{m}+\Lambda_{i}\left(t-\tau \right)S_{i}\left(t-\tau \right)-\mu_{i}I_{i}-fI_{i}\text{,}$$

$${{\dot{S}}_{i}}^{m}=fS_{i}-r{S_{i}}^{m}-\Lambda_{i}{S_{i}}^{m}-\mu_{i}{S_{i}}^{m}\text{,}$$

$${{\dot{E}}_{i}}^{m}=fE_{i}+\Lambda_{i}{S_{i}}^{m}-r{E_{i}}^{m}-\Lambda_{i}\left(t-\tau \right){S_{i}}^{m}\left(t-\tau \right)-\mu_{i}{E_{i}}^{m}\text{,}$$

$${{\dot{I}}_{i}}^{m}=fI_{i}+\Lambda_{i}\left(t-\tau \right){S_{i}}^{m}\left(t-\tau \right)-r{I_{i}}^{m}-\mu_{i}{I_{i}}^{m}$$

The subscript i denotes the mosquito species, β is the number of emerging adult mosquitoes (unmarked, taking a different value for each site), and Λ represents the force of infection from humans to mosquitoes. All parameter definitions and values are provided in Supplementary Table S1. As the parasite undergoes an incubation period within the vector before the mosquito becomes infectious, the rate of entry into the infectious compartments depends on the number of susceptible mosquitoes and the force of infection from τ timesteps in the past as follows

(2) $$\mu_{i}=\mu_{i}^{nat}+\mu_{i}^{\mathrm{int}}+\mu_{i}^{dye}$$

where $\mu_{i}$ denotes the species-specific daily mortality and consists of natural mortality ${\mu_{i}}^{nat}$, intervention induced mortality ${\mu_{i}}^{\mathrm{int}}$, and any dye-induced mortality ${\mu_{i}}^{dye}\text{.}$ Daily bait-feeding rates were estimated numerically by adjusting the daily bait-feeding rate, f, in the ASB model until the proportion of dyed mosquitoes matches the desired or observed dyed fraction. Values are estimated independently for each scenario by using site-specific parameters such as seasonality in mosquito population dynamics, ITN usage, pyrethroid resistance and dye-related parameters such as dye longevity and dye-induced mortality. Other experiment-related parameters such as the timing of mosquito collections in relation to ASB deployment are explicitly captured. Uncertainty in the bait-feeding rate estimates are generated by conducting a multivariate sensitivity analysis on ASB input parameters using the parameter ranges outlined in Supplementary Table S1. Sensitivity analysis is used to highlight the relationship of various parameters to ATSB efficacy but is unlikely to represent the full uncertainty expected from all parameters and model assumptions (Kleijnen, 1994).

### 2.2 Individual-based transmission model

The study extends an existing individual-based mathematical model of *Plasmodium falciparum* transmission dynamics to assess the impact of ATSB stations on both entomological and epidemiological outcomes. This model, previously outlined in detail (Griffin et al., 2010, 2014; White et al., 2011), delineates the life cycle dynamics of malaria transmission. In brief, individual people are born susceptible, initially shielded by maternal immunity which diminishes within the first six months of life. Susceptibility to infection arises from exposure to mosquito bites, influenced by factors such as mosquito abundance, infectivity, and the age of humans. Infected individuals may manifest clinical symptoms or remain asymptomatic. Those with clinical symptoms may undergo treatment, clearing the parasite and entering a prophylactic phase before reverting to susceptibility. A portion of infected individuals may remain untreated, eventually clearing the disease and transitioning to an asymptomatic state. Asymptomatic infections later regress to a sub-patent stage and finally revert to susceptibility. Throughout these states, individuals remain susceptible to reinfection. The human model is individual-based and stochastic whilst the mosquito model is compartmental and deterministic, tracking the mean number of susceptible S, exposed E, and infectious I mosquitoes per person as outlined in the ASB model, though excluding the dyed life-stages. Following previous studies (Marshall et al., 2013; Fraser et al., 2021) it is assumed that ATSBs act by increasing the mosquito daily mortality rate. It is also assumed that the bait-feeding rate derived from ASB experiments using the deterministic ASB model equates to the surplus mortality that would have been observed had ATSBs been implemented in those communities (i.e. we assume that all mosquito bites on ATSB stations are fatal). Mosquito mortality now comprises natural mortality, mortality stemming from interventions other than ATSBs, and ATSB mortality during periods of ATSB deployment (${\mu_{i}}^{ATSB}$) as follows

(3) $$\mu_{i}={\mu_{i}}^{nat}+{\mu_{i}}^{\mathrm{int}}+{\mu_{i}}^{ATSB}$$

Unless otherwise stated all parameters are taken from the default conditions defined in Churcher et al. (2024). This paper builds on both previous modelling studies of ATSB by calculating the expected bait-feeding rate using field data from wild-caught mosquitoes. Additionally, it extends the work of Fraser et al. (2021) by explicitly modelling ITN and IRS campaigns as time-dynamic processes following a framework that has been validated against data from cluster RCTs (Sherrard-Smith et al., 2018; Nash et al., 2021). All human components of the model follow previous work (Griffin et al., 2010), with different simulations adjusting the human-mosquito ratio to vary the baseline disease endemicity.

### 2.3 Intervention scenarios

#### 2.3.1 ATSB scenarios

In accordance with previous modelling studies, we initially treat ${\mu_{i}}^{ATSB}$ as a daily mortality rate which affects the whole mosquito population equally, making the assumption that bait station feeding rates are independent of the blood-feeding rate (which itself varies according to the use of vector control) and mosquito age (i.e. mosquitoes bait feed at a constant rate throughout their lifetime). It is initially assumed that the sampling of mosquitoes caught in ASB studies is unbiased (i.e. caught mosquitoes are a true representation of the mosquito population transmitting malaria) though this assumption is then relaxed, to consider how epidemiological impact might vary according to these three scenarios:

(i) A scenario in which mosquitoes fed on bait stations may be more or less likely to be caught than unfed mosquitoes. This was achieved by multiplying the true feeding rate by a factor representing the degree of overrepresentation within the ASB-caught mosquitoes relative to the total mosquito population. For example, if fed mosquitoes are twice as likely (or unlikely) to be caught, the feeding rate was multiplied by 2 (or divided by 2) leading to 100% overestimation (or underestimation). This could be the case if traps are placed in close proximity to bait stations. This scenario can also be interpreted as the effect of reducing the lethality of ATSB because a 100% overestimation of bait-feeding is equivalent to a lethality rate of 50%, for example.
(ii) A scenario in which bait-feeding occurs daily but only in a subset of mosquitoes. This was achieved by creating two identical species which differ only in their propensity to feed on the bait stations. The fraction of the total population which feeds on bait stations before the onset of ATSBs and to which ${\mu_{i}}^{ATSB}$ is applied is called the exposed fraction.
(iii) A scenario in which the whole population is liable to bait-feeding, but less frequently than daily. This was achieved by including a step function that allows ${\mu_{i}}^{ATSB}$ to apply once every three days, twice every three days, or thrice every three days (daily feeding). This assumes that mosquitoes only have the opportunity to be caught in traps on days in which they are able to feed on bait stations. This may be the case if mosquitoes are only exposed to traps and bait stations on days when they enter a village seeking a blood meal, while on other days they find sugar meals outside the village where there is no opportunity to feed on bait stations or be caught in traps.

In the former two scenarios, we consider both the entomological effect that ATSBs would have on the whole population (unbiased sampling) and the effect they would have on the population which are exposed to ATSBs (biased sampling).

#### 2.3.2 ITN, IRS and SMC scenarios

The impact of ATSBs will depend on historical and future malaria control. To evaluate this impact across various potential settings, we consider five scenarios.

(a) No control: a scenario with no malaria prevention activities. This illustrates the impact ATSB stations could have on their own to allow a comparison with existing control interventions.
(b) Pyrethroid-only ITNs: historical pyrethroid-only ITNs followed by a mass distribution of pyrethroid-only ITNs at time 0.
(c) Pyrethroid-pyrrole ITNs: historical pyrethroid-only ITNs followed by a mass distribution of pyrethroid-pyrrole ITNs at time 0.
(d) SMC: historical pyrethroid-only ITNs until time 0 followed by seasonal malaria chemoprevention (SMC) administered in four rounds every year to children aged 3 months to 5 years starting at time 0.
(e) IRS: historical pyrethroid-only ITNs until time 0 followed by IRS every year starting at time 0.

Simulations are run for each scenario with and without ATSB stations (the latter being equivalent to a bait-feeding rate of zero) and the difference in the total number of clinical cases in the population from these two sets of simulations is provided as an estimate of the number of cases averted.

To allow direct comparisons, we assume a default deployment time of six months following a large-scale implementation in Mali (Traore et al., 2020) and a baseline entomological inoculation rate (EIR) of 52 infectious bites per person per year across as the default parameterization unless otherwise specified. This corresponds to a 40% prevalence given 20 years of routine pyrethroid-only ITN use. Historical ITN usage estimates were taken for Keyes Province, Mali, and Western Province, Zambia, from maps generated by the Malaria Atlas Project (Bertozzi-Villa et al., 2021, malariaatlas.org). Future ITN mass distributions with either pyrethroid-only or pyrethroid-pyrrole nets were assumed to achieve 70% population usage immediately after the mass campaign (with each net being used for an average of 2.5 years), SMC campaigns were assumed to reach 80% of children aged 3 months to 5 years, and IRS campaigns were assumed to cover 40% of the population. In each scenario, we investigate the effect of combining ATSBs with the intervention strategy already in place. All parameters and the evidence-base for the intervention effectiveness are provided in Supplementary Table S1. These default parameters were used across all simulations unless otherwise specified.

Simulations represent the mean of 10 runs, with uncertainties reflecting parameter variation within bounds specified in Supplementary Table S1. Upper and lower uncertainty bands are calculated using the corresponding parameter limits averaged across 10 simulations.

### 2.4 Data

Dyed fraction measurements were taken from ASB studies undertaken in Mali and Zambia. In the Keyes Province of Mali, a field experiment was conducted in 14 different clusters from April to December 2016 (Traore et al., 2020). Half of the clusters were visited each month and two ASB stations containing a green food dye were hung on every house in the cluster and mosquitoes were caught until the following day using 10 CDC UV light traps that were placed 5 m away from each house. The following year, ATSB stations were deployed in the remaining clusters and entomological surveillance continued monthly to assess mosquito abundance using CDC light traps.

In the Western Province of Zambia, a study was conducted in 10 study clusters to assess dyed fraction (Chanda et al., 2023). ASB stations with uranine dye were hung on eligible structures in February 2021. Dyed fraction measurements were made during two 6-week periods using CDC light traps which were placed a minimum of 5–10 m away from any ASB but with half the traps inside the structure. ASB stations were removed in early June 2021.

### 2.5 Comparisons to observed mosquito catch

To explore the ability of the model to predict changes in adult mosquito abundance we used Mali as a case study as it is the only site which currently has dyed fraction (ASB) data alongside measures of mosquito abundance during ATSB implementation. As ASB stations were only left out for one night in Mali, with the proportion of dyed mosquitoes being measured the following day, we assumed that the dyed fraction measured in this experiment was equal to the daily bait-feeding rate. Total mosquito abundance and seasonality forecasted by the model is translated to cluster-level abundance estimates for the control clusters by fitting a scaling factor to minimise the sum of squared differences between the observed and model-predicted abundances. The ATSB transmission dynamics model with the estimated bait-feeding rate is used to predict the relative difference in mosquito counts between the control and ATSB arms. Model projections are compared with the observed catch data from the trial using the mean squared deviation ratio (MSDR) relative to a baseline and *R*^2^. To calculate MSDR we compared the model estimates to a baseline model consisting of the mean of the observed values.

## 3 Results

### 3.1 Deploying ATSB stations with standard malaria control

**Fig. 2:**
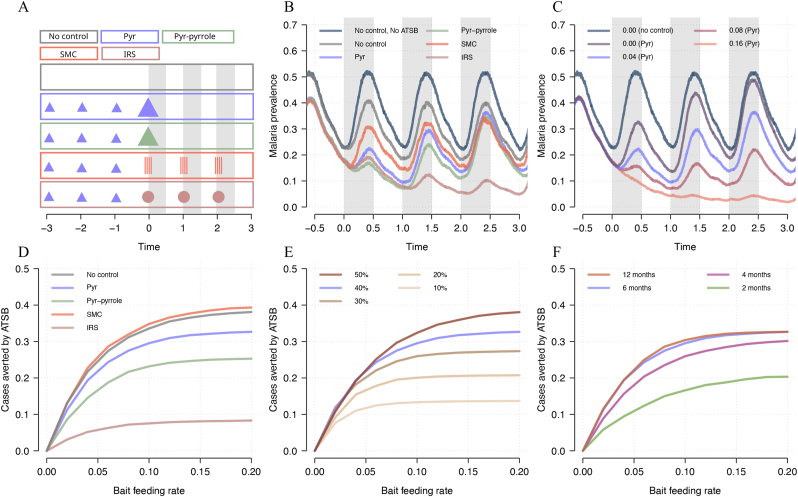
Entomological and epidemiological impact of ATSB. **A** Diagram of intervention scenarios denoting control before and after ATSB was introduced at time 0. **B** Model projections for parasite prevalence over time in 6 months to 25 year-olds over time for deploying ATSBs on different interventions at a fixed feeding rate of 0.04 per day. **C** Projections of different ATSB feeding rates in combination with pyrethroid-only nets. **D** All-age clinical malaria cases averted per person year relative to no ATSB in combination with different interventions, varying malaria endemicity (**E**), and the effect of varying the period of ATSB deployment per year (**F**). In panel **A**, triangles indicate an ITN mass distribution, circles indicate an IRS campaign, and each vertical line indicates one round of SMC. Small symbols indicate annual programmes whilst large triangles denote a single mass campaign, after which nets are lost. Grey vertical shaded areas in **A**-**C** indicate the default period of ATSB station deployment. In panels **B** and **D**, ITN distributions are assumed to reach 70% population usage, IRS campaigns are assumed to reach 40% of the population and SMC is assumed to reach 80% of children aged 3 months to 5 years. In panel **E** baseline prevalence was achieved by setting the starting EIR to 114, 52, 23, 11.5, and 5.5 infectious bites per person per year. Panel **F** shows the effect of varying the period of ATSB deployment. Estimates for the relative reduction in the number of cases are provided in Supplementary Fig. S1.

Models project that if the whole mosquito population is liable to feed regularly on bait stations, then ATSBs are projected to have a substantial impact on malaria prevalence (Fig. 2B and C) and mosquito density (Supplementary Figs. S1B and C). Projections indicate that the level of standard malaria control already in use affects the absolute impact of ATSBs. For a given feeding rate, ATSBs avert fewer cases in regions where vector control interventions are already working efficiently, though the relative reduction in cases may be larger. For example, ATSBs cause smaller reductions in malaria prevalence in the first year of an ITN campaign than the third year, when fewer people are using ITNs which are older and provide less effective vector control (Fig. 2C). Similarly, ATSBs perform less well when deployed together with highly effective interventions such as IRS in contrast to introducing them alongside comparatively less effective interventions such as pyrethroid-only ITNs in areas with pyrethroid resistant mosquitoes (Fig. 2D). This is driven by the non-linear relationship between changes in mosquito mortality and the epidemiological benefit this causes (Fig. 2D and E, Supplementary Fig. S1A). Small perturbations to the feeding rate close to zero have a disproportionately stronger effect compared to the same perturbation at a higher feeding rate and when the feeding rate reaches 0.16, while many mosquitoes are still alive (Supplementary Fig. S1C), relatively few live long enough to contribute infectious bites to the EIR (Supplementary Fig. S1A). For example, in a moderate transmission site with pyrethroid-only ITNs, increasing the bait-feeding rate from 0.00 to 0.05/day is predicted to reduce clinical incidence by 0.22 cases per person, whereas going from 0.05 to 0.10/day is only expected to avert an additional 0.08 cases per person. ATSBs are predicted to avert more cases in areas of higher transmission (Fig. 2E) and if it is used throughout the year, though in highly seasonal areas targeted use for shorter periods during the peak of the transmission season is predicted to have substantial benefit (Fig. 2F).

**Fig. 3:**
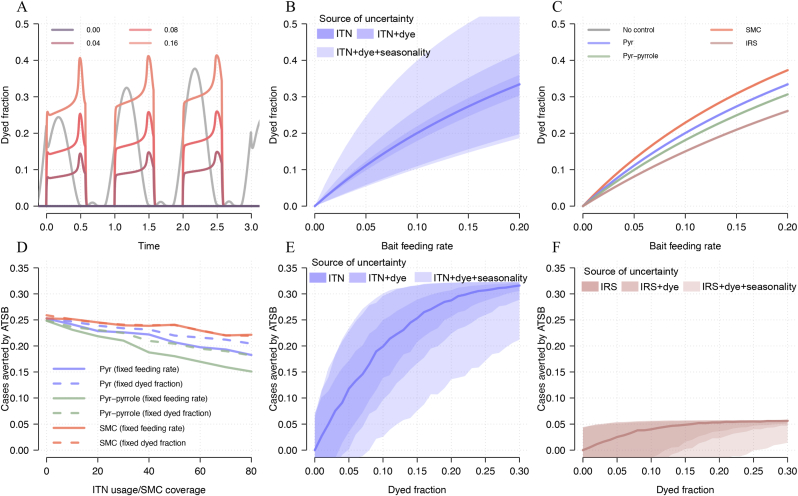
Estimating daily feeding rate in the presence of interventions. Dyed fraction dynamics over time for various feeding rates in combination with pyrethroid-pyrrole nets (**A**) and dyed fraction as a function of feeding rate (**B**, **C**). Cases averted per person year as a function of intervention coverage (**D**) and as function of dyed fraction for the pyrethroid-only ITN scenario (**E**) and the IRS scenario (**F**). The light grey line in panel **A** shows the seasonal mosquito abundance on an arbitrary scale. In panels **B** and **E** ITN uncertainty consists of allowing ITN usage to vary from 50 to 90% and population level pyrethroid resistance to vary from 50 to 90%; dye uncertainty consists of allowing dye longevity to vary from 2.1 to 7 days and dye mortality to vary from 0 to 10%; seasonality uncertainty consists of allowing the sampling day to vary from 10 to 180 days post bait deployment. In panel **C**, ITN campaigns were assumed to reach 70% of the population, IRS was assumed to reach 40% and SMC was assumed to reach 80% of 3 month to 5 year olds. In **D**, the dashed lines represent a fixed feeding rate of 4% and the solid lines represent a fixed dyed fraction of 10.7% (which corresponds to 4% feeding assuming no interventions, but where the feeding rate varies according to other control). Note that both lines overrun each other for SMC as this intervention does not change the relationship between ASB dyed fraction and feeding rate. In **F**, IRS uncertainty consists of allowing IRS coverage to vary from 20 to 60% with dye and seasonality uncertainty having the same definition as in panels **B** and **E**. Estimates for the relative reduction in the number of cases are provided in Supplementary Fig. S3.

If ATSBs are deployed at scale, then the feeding rate is likely to be unknown. In this situation the level of vector control will also affect the impact of ATSBs by influencing the feeding rate (given the observed dyed fraction). For instance, when the feeding rate is fixed, the dyed fraction in the third year of an ITN campaign will likely be higher than if it was measured in the first year, as more people will be using ITNs in the first year causing higher mosquito mortality (Fig. 3A). Dyed fraction is highly dependent on seasonality in mosquito abundance, with dye positivity peaking at the end of the transmission season as the average age of the mosquito population increases. Similarly, any given dyed fraction will correspond to a higher feeding rate if it was measured in an area with more effective vector control such as IRS or pyrethroid-pyrrole ITNs (Fig. 3C). For example, measuring a dyed fraction of 0.20 would correspond to a feeding rate of 0.14/day if measured in an area with IRS, 0.12/day if measured where people are using pyrethroid-pyrrole ITNs, 0.10/day if measured in an area with pyrethroid only ITNs or 0.08/day if no vector control tools are in use (Fig. 2C) based on continuous availability of ASB stations and a mean dye longevity of 4.5 days. Note that the level of SMC use does not impact the relationship as it is assumed to have no direct effect on the life-expectancy of the vector population.

As vector control interventions influence the expected feeding rate in a way that other interventions do not, we explored the effect of closing the feedback loop between intervention coverage, dyed fraction and the feeding rate. Our results show that for a given observed dyed fraction, increasing ITN usage leads to a reduction in the epidemiological impact of ATSBs but one that is slower than would be expected if the feeding rate remained constant; an effect which is not observed for non-vector control interventions (Fig. 3D). This is because the same observed dyed fraction in the presence of higher ITN usage leads to increased overall mosquito mortality and therefore leads us to predict a higher feeding rate for the same dyed fraction (all else being equal). This means that the effectiveness of ATSBs diminishes less with increased use of ITNs given an observed dyed fraction compared to what would be expected if the feeding rate remained the same (Fig. 3D).

### 3.2 Estimation of ATSB station impact from dyed fraction alone leads to uncertainty

The relationship between dyed fraction and feeding rate is influenced not only by the vector control interventions already in place, but also by the properties of the dye used to stain mosquitoes and the sampling technique used. The ASB model suggests that a site where the true feeding rate is 0.10/day could observe dyed fractions between 10 and 40% in settings with uncertain net usage (50–90%), pyrethroid resistance (50–90%), dye longevity (2.1–7.0 days), dye mortality (0–10%) and sampling approaches (early-late in the season, Fig. 3B) leading to drastically different epidemiological outcomes (Fig. 3E). This uncertainty is influenced by alternative vector control use (less effective interventions lead to lower feeding rates), the longevity of the dye used to stain mosquitoes (longer longevity leads to lower feeding rates), any excess mortality induced by the dye (lower dye mortality leads to lower feeding rates), and when in the season sampling takes place (sampling earlier when population is growing leads to lower feeding rates). A more detailed analysis of how ITN use, pyrethroid resistance, dye mortality and longevity contribute to this uncertainty is provided in Supplementary Fig. S2.

Uncertainty in the feeding rate also leads to uncertainty in epidemiological outcomes. For example, a dyed fraction of 0.10 measured in a setting with pyrethroid-only ITNs could imply a public health benefit of anywhere between 0.03 and 0.30 clinical cases averted per person per year (Fig. 3E). Measuring the same dyed fraction in a setting where IRS is occurring leads to even greater uncertainty due to the efficacy of this intervention varying substantially according to population coverage (Fig. 3F). Conversely, in a setting with no alternative vector control the outcome is projected to be less uncertain (Supplementary Fig. S3C). Using the full range of uncertainty shown in Fig. 3B to translate cluster level species-specific dyed fractions into bait-feeding rates, we project potential outcomes of ATSBs on malaria in Western Province, Zambia, showing the wide range of uncertainty in malaria prevalence generated (Supplementary Fig. S4). This uncertainty could be considerably reduced by measurement of standard vector control coverage during the ASB feeding study.

### 3.3 Model projections are broadly consistent with entomological data

**Fig. 4:**
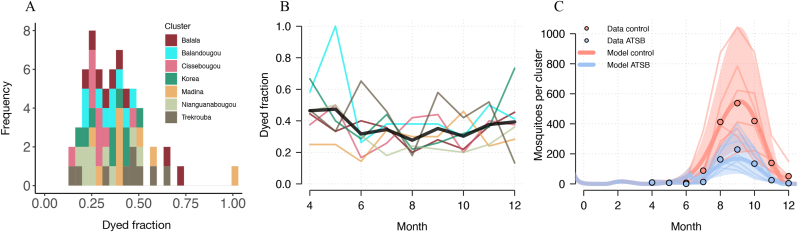
Model results reproduce entomological impact of ATSB in Mali. Dyed fractions broken down by cluster (**A**) and time (**B**, with the thick black line indicating the mean estimate for that timepoint). **C** Predictions of the ability of ATSB to change mosquito abundance given the observed ASB dyed fraction and the mean number of mosquitoes caught in the control arm. Model predicted mean (*thick line*) and uncertainty (*shaded region*) is compared to reported mosquito abundances by intervention arm. The faint lines correspond to observed individual cluster trajectories. The shaded region represents the 5th and 95th quantiles of data generated by repeatedly sampling a random net usage and pyrethroid resistance of 50–90% and a random cluster level mosquito density and cluster level dyed fraction.

A wide range of dyed fractions were measured in Mali (mean 0.34, standard deviation 0.15), varying both within and between clusters (Fig. 4A). Although cluster level dyed fractions fluctuate month on month, the mean for the period during which ATSB stations were deployed (June-December) was stable (Fig. 4B). The entomological collections from CDC light traps showed a decrease in trapped mosquitoes of 57% in ATSB clusters relative to control clusters. By fitting to the mosquito density in control clusters and using the reported dyed fractions, the model can broadly reproduce the mosquito density observed in ATSB clusters (MSDR = 0.15, *R*^2^ = 0.90, Fig. 4C).

### 3.4 Projections are highly sensitive to bait-feeding behaviour

**Fig. 5:**
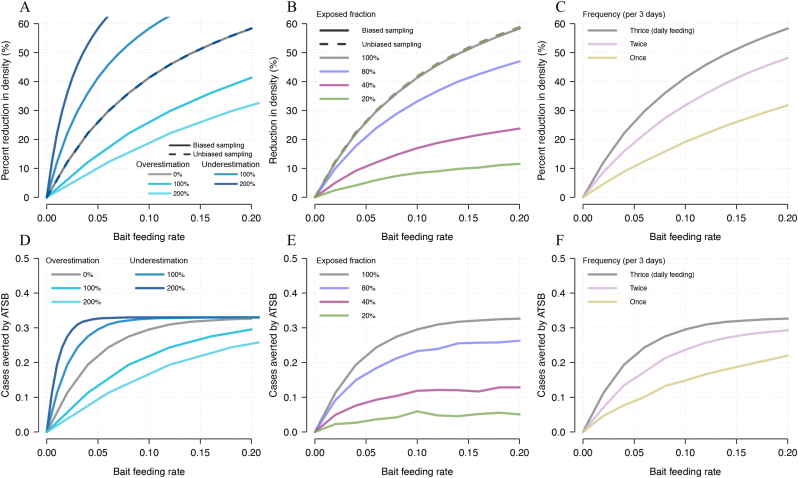
Model projections of the effect of entomological sampling bias and uncertainties in sugar-feeding behaviour on the entomological and epidemiological impact of ATSBs. Model predicted estimates for percentage reduction in mosquito density (**A-C**) and absolute number of cases averted per person-year (**D-F**) as a function of observed bait station feeding rate (as defined as the percentage of mosquitoes feeding per day). Note that the observed bait-feeding rate (which can be estimated from the dyed fraction of mosquitoes caught in traps) will be different from the true bait-feeding rate in the overall vector population in all simulations other than the default (which assumes both observed and true are the same). Different plausible model assumptions are relaxed and compared to the default model. Panels **A** and **D** show different levels of bait-feeding rate overestimation, be it no bias (default model, *dark blue line*) or bias which could be caused by stained mosquitoes more likely to be caught than those transmitting malaria. Panels **B** and **E** show varying proportions of the mosquito population which feed on bait stations, from 100% (no heterogeneity in mosquito population, default model, *red line*) to only 20% of the mosquito population feeding on bait stations (and caught in ASB traps) with the other 80% not feeding on bait stations (and not being caught in traps, *green line*). Panels **C** and **F** show models which assume that the observed feeding rate happens every day (default model, *orange line*), twice every three days (*yellow line*) or once every three days (*turquoise line*). For entomological outcomes, both the effect on the total population (unbiased sampling) and in the sampled population (biased sampling) are shown in solid and dashed lines, respectively. All dashed lines overrun each other, as the estimated feeding rate in the sampled population is shown on the x-axes. Estimates of the relative reduction in the number of cases are provided in Supplementary Fig. S5.

The considerable uncertainty in the epidemiological impact of ATSBs (Fig. 3E and F) is further compounded by uncertainties in the reliability of the dyed fraction estimates and the biology of mosquito sugar-feeding behaviour. Up to now, it has been assumed that the proportion of mosquitoes caught with dye is a true representation of the total mosquito population transmitting malaria, i.e. that a mosquito that has fed on a bait station is just as likely to be caught as any other. However, if traps are biased and preferentially catch stained mosquitoes then the dyed fraction measured will lead to an overestimation of the true population feeding rate, overestimation of the entomological impact (Fig. 5A) and cases that could be averted by ATSBs (Fig. 5D). For example, if traps are preferentially catching sugar-feeding mosquitoes in the vicinity of ASBs then the dyed fraction could be higher than those blood-feeding who might be less likely to be caught in a trap. Note that if the same trapping biases are seen for estimates of mosquito density in ATSB studies (as seems likely in Mali as the same trapping methodology was used) then the model could reliably predict differences in mosquito abundance in the sampled population but could fail to predict changes in the total vector population and hence fail to capture epidemiological impact. For example, if the observed bait-feeding rate was 0.050 per day, then ATSBs are projected to reduce mosquito abundance by 26% and reduce malaria clinical incidence by 0.23 cases per person per year. If the entomological collections were twice as likely to catch mosquitoes that had bait fed than the average vector in the population then the true feeding rate would be 0.025 per day (2.5%) the reduction in mosquito abundance in the overall mosquito population would be 15%, though the observed reduction in the traps would remain at 27%. In this case the traps would measure a reduction in mosquito abundance, but the intervention would be predicted to elicit less of an epidemiological benefit, averting a projected 0.13 cases per person per year.

Secondly, while it is thought all mosquitoes require sugar meals, there are many potential sources of sugar and it may not be the case that all mosquitoes will take a sugar meal from a bait station. The measured dyed fraction could, therefore, be the result of a sub-population of mosquitoes which feed on bait stations whilst the rest of the population seeks sugar meals elsewhere. Our default model assumes that all mosquitoes have an equal probability of feeding on bait stations, but if there is heterogeneity in the mosquito population, and some mosquitoes maintain a lower probability of feeding on baits than others, then those not feeding on bait stations could sustain transmission. As the size of the exposed fraction of mosquitoes falls, the public health benefit of ATSBs also falls dramatically (Fig. 5B). Like before, if the same traps are used for entomological assessments, the density reduction in the exposed population will overestimate the expected population level reduction and epidemiological impact (Fig. 5B-E).

Finally, in line with the previous modelling studies on ATSBs, we have assumed that bait-feeding occurs daily (Marshall et al., 2013; Fraser et al., 2021). If mosquitoes do not feed on sugar daily or are not exposed to ATSB stations daily but are only in a position to feed on bait stations once or twice every three days, the potential impact of ATSBs is considerably diminished (Fig. 5C-F) assuming that the mosquitoes can only be caught on days when they are seeking to feed on ASB stations.

## 4 Discussion

The modelling framework presented in this study can use the dyed fraction of mosquitoes from ASB studies to broadly reproduce the entomological impacts of ATSBs. It indicates that if ATSBs can be successfully implemented at scale, it could potentially prevent large numbers of clinical cases of malaria, though estimation of the epidemiological impact of ATSBs using dyed fraction alone is highly uncertain. ASB studies in Mali and Zambia show very high variability in the dyed fraction of mosquitoes, which varies considerably between clusters and over time. This uncertainty in the dyed fraction leads to very high uncertainty in the bait-feeding rate, driven by uncertainty in the conversion between dyed fraction and feeding rate, the presence of vector control interventions, the seasonality of mosquito bionomics and the properties of the ASB dye. The uncertainty in bait-feeding rates will depend upon the ASB study design. While ATSBs have shown a potential to significantly reduce the number of mosquitoes caught, our results highlight that this may not necessarily lead to a large public health benefit depending on the bias in the sampling of mosquitoes and their bait-feeding behaviour.

### 4.1 Combining ATSBs with standard malaria control

This work indicates the mass deployment of ATSBs can be considered alongside existing control interventions, but that its efficacy could vary according to which other tools are in use. All else being equal, ATSBs are predicted to have a smaller epidemiological benefit in terms of absolute reduction in the number of cases averted when combined with more effective vector control (Fig. 1D), though relative reductions are generally more consistent (Supplementary Fig. S1D). This is in line with previous empirical work which suggests modest benefits of adding a single round of IRS the same year as an ITN mass campaign (Protopopoff et al., 2018). Theory about the relationship between intervention coverage, the reproduction number and vectorial capacity indicates that the relationship between mosquito mortality and the basic reproduction number is highly non-linear (Brady et al., 2016). The extent to which this influences the number of clinical cases will depend on the setting and differences in human immunity. Typically, in areas with higher malaria transmission, more cases can be averted, while subsequent interventions provide diminishing returns. When the bait-feeding rate is uncertain, models suggest a more complex scenario for ATSBs. Vector control measures like ITNs and IRS reduce the dyed fraction in ASB studies, meaning that sites with effective vector control but similar dyed fractions likely have a higher bait-feeding rate, boosting ATSB's projected impact. This increased bait-feeding rate does not fully offset the reduced number of cases averted by ATSBs when ITN use is high, highlighting the need to understand how different factors interact in ASB studies. Models indicate that ATSBs avert the same number of cases whether or not it is used alongside SMC, in part because interventions which target humans do not influence mosquito mortality which is non-linearly related to intervention impact (Brady et al., 2016).

### 4.2 Lethality of ATSBs

A significant challenge facing all vector control tools is the increasing threat posed by insecticide resistance with pyrethroid resistance in at least one malaria vector being reported in 87% of the countries which reported data to the World Health Organization (WHO, 2023). While the dinotefuran used in ATSBs is a neonicotinoid, a class of insecticide not used in ITNs, there is already potential for resistance to have emerged in malaria vectors due to the widespread use of neonicotinoids in agriculture, and their subsequent leakage into the environment and non-target organisms (Okeke et al., 2024). Aside from this, continued use of dinotefuran in ATSB stations could also provide selective pressure for neonicotinoid resistance to emerge among malaria vectors in the same way that resistance to pyrethroids has increased following the widespread use of ITNs. Careful monitoring of dinotefuran resistance is therefore needed should the intervention be adopted more widely.

The models initially assume 100% lethality for all mosquitoes feeding on ATSBs based on laboratory studies which indicate that ingestion of dinotefuran concentrations of 1 ng were sufficient to kill susceptible (Kisumu) strain of *An. gambiae* (*s.s.*), which is much smaller than observed meal sizes when provided free access to dinotefuran sugar solution. Nevertheless, this assumption could be overly optimistic and could oversimplify the real-world dynamics. In practice, lethality could vary due to factors such as insecticide resistance. Furthermore, the assumption that ATSB stations do not experience a decay in lethality over time contrasts with the modelled decay in the efficacy of ITNs and IRS. The models presented here can be used to explore scenarios with lower ATSB lethality, for example in Fig. 2F, where we vary the deployment duration of ATSB stations or in Fig. 5A-D, where overestimation of the bait-feeding rate serves as a proxy for lower lethality (i.e. an overestimation of the bait-feeding rate by 100% or 200% is equivalent to an ATSB lethality rate of 50% and 25%, respectively).

### 4.3 Sugar-feeding behaviour

Little is known about how the natural sugar-feeding behaviour of mosquitoes translates to feeding on sugar baits. Previous ASB feeding studies have found high dyed fractions which in some settings have exceeded 30% (Traore et al., 2020). This seems to indicate that a significant proportion of the mosquito population could be eliminated should ATSBs be introduced and therefore considerable reductions in malaria could be expected (Fraser et al., 2021). These models make a variety of assumptions which could influence model reliability. For example, previous work has assumed that a mosquito has a similar probability of feeding on bait each day and that the whole mosquito population is liable to take sugar meals from bait stations leading to the conclusion that impressive entomological and epidemiological outcomes can be expected from even relatively low feeding rates (Marshall et al., 2013; Fraser et al., 2021). In our study, we show how this may not be the case in certain scenarios such as if bait-feeding occurs once or twice per gonotrophic cycle instead of daily, if a fraction of the mosquito population never feeds on bait, or if bait-fed mosquitoes are more likely to be caught in traps than other mosquitoes (Fig. 5). There are a range of additional hypotheses not explored here that could reduce the impact of ATSB and maintain high levels of transmission. One such hypothesis is if there were a negative interaction between sugar-feeding and blood-feeding. There is evidence to show that blood-feeding and sugar-feeding behaviour interact such that blood-feeding is lower in mosquitoes which have access to sugar (Straif and Beier, 1996) and sugar-feeding is lower in mosquitoes that have blood-fed (Omondi et al., 2022). Both *An. gambiae* and *Aedes aegypti* females can forego sugar-feeding and instead take multiple blood meals during a gonotrophic cycle (Scott and Takken, 2012) and *Plasmodium* infection itself may bias mosquito behaviour towards blood-seeking through altering olfactory responses (Stanczyk et al., 2019), which may make mosquitoes less likely to seek a sugar meal. Therefore, it may be that the mosquitoes which enter a village seeking a sugar meal are killed by ATSB stations but that these do not represent the population that is maintaining the majority of transmission. There is therefore a need to understand whether there is a difference between the mosquitoes being sampled in traps and those that are contributing to transmission.

Our results show that ATSB's impact can be attenuated if mosquito bait-feeding occurs less frequently than daily. Although little is known about the frequency of natural sugar-feeding in anophelines, one study has found that, while male *An. gambiae* may sugar feed twice per night on average, females with access to blood and oviposition sites only sugar fed once in four nights (Gary and Foster, 2006). Furthermore, even if sugar-feeding in females was a daily behaviour, they may take sugar meals from natural sources before they have the opportunity to take one from a bait station. Alternatively, the bait-feeding rate could change with mosquito age, which would reduce the impact of ATSBs if older mosquitoes, which are more likely to be infectious, are feeding less frequently. This may not be evident from current ASB studies which will catch primarily young mosquitoes even if the probability of being caught in a trap is independent of mosquito age. Our work also assumes that bait-feeding rate is constant throughout the transmission season. There is no clear seasonality in the dyed fraction in the ASB studies in Mali and Zambia, though this could be due to the high variability in caught mosquitoes. Further work is needed in a more diverse range of settings as bait-feeding rate might change throughout the season as the availability of alternative sugar sources varies.

### 4.4 Trapping bias

Our results indicate that trapping bias can affect both the estimation of the feeding rate and the estimation of entomological outcomes (Fig. 5). Bias in a variety of trapping methods has been extensively documented, and a recent review concludes that using different trapping methods in the same site rarely produces consistent results (Degefa et al., 2024). For example, out of two studies of *An. funestus* in Kenya which recorded mosquito densities, the ratio of mosquitoes caught from CDC light traps compared to human landing catch was 1.91 in one study (Mathenge et al., 2005) and 0.69 in the other (Wong et al., 2013). CDC light traps, used in both ASB feeding studies, have been reported to be less reliable for catching outdoor host-seeking anophelines (Overgaard et al., 2012; Kenea et al., 2017) and their ability to trap mosquitoes consistently over their lifetime, not only during the host-seeking period which is needed to assess sugar-feeding rates, is highly unclear. In addition, the traps in the ATSBs field trial in Mali and Zambia were placed within 5–10 m of a structure with a bait station, so might have experienced a higher than expected local dyed fraction, resulting in a larger reduction in mosquito density not observed across the whole region (Traore et al., 2020). Together, this suggests that trapping bias may overestimate reductions in mosquito density both due to proximity to ATSBs and due to bias against host-seeking mosquitoes. If the trapping methods used are sampling the same population that is exposed to bait stations (the exposed fraction), we can expect to see a smaller impact on malaria epidemiology even in the face of impressive observed reductions in mosquito abundance (Fig. 5).

## 5 Conclusions

ATSBs have the potential to be an effective method of controlling malaria alongside existing control interventions. Overall, our results show that forecasting ATSB efficacy using dyed fraction alone is possible but highly uncertain and can be improved with better information about mosquito population dynamics, background vector control, dye properties and the sampling approach of ASB studies. Model estimates can broadly reproduce entomological results observed when ATSBs are deployed at scale, but the full epidemiological impact is unclear and can be obscured if sampling methods are biased, leading to overestimation of the feeding rate and entomological impact. Further studies to better understand the sugar- and bait-feeding behaviour of mosquitoes are needed to better understand the potential of this novel method of delivering vector control.

## Ethical approval

Not applicable.

## CRediT authorship contribution statement

**Nima R. Moghaddas:** Conceptualisation, Methodology, Software, Formal analysis, Investigation, Visualisation, Writing – original draft, Writing – review & editing. **Mohamed M. Traore:** Data curation, Writing – review & editing. **Günter C. Müller:** Data curation, Writing – review & editing. **Javan Chanda:** Data curation, Writing – review & editing. **Joseph Wagman:** Data curation, Writing – review & editing. **Julian Entwistle:** Conceptualisation, Writing – review & editing. **Christen M. Fornadel:** Conceptualisation, Writing – review & editing. **Thomas S. Churcher:** Supervision, Conceptualisation, Methodology, Writing – review & editing, Funding acquisition. All authors read and approved the final manuscript.

## Funding

This study was funded by IVCC through support from the Bill & Melinda Gates Foundation (grant: INV-007509), the Swiss Agency for Development and Cooperation (SDC) (grant: 81067480) and UK Aid (grant: 30041-105). The findings and conclusions contained within are those of the authors and do not necessarily reflect the positions or policies of the Bill & Melinda Gates Foundation, SDC, UK Aid or IVCC. Nima R. Moghaddas and Thomas S. Churcher acknowledge funding from the Medical Research Council (MRC) Centre for Global Infectious Disease Analysis (reference number MR/R015600/1), jointly funded by the MRC and the UK Foreign, Commonwealth and Development Office (FCDO), under the MRC/FCDO Concordat agreement, and is also part of the EDCTP2 programme supported by the European Union.

## Declaration of competing interests

The authors declare that they have no known competing financial interests or personal relationships that could have appeared to influence the work reported in this paper.

## Data Availability

The data used in this study can be found in Traore et al. (2020) and Chanda et al. (2023).
