# Supplementary material for: Exploring the epidemiological impact of attractive targeted sugar bait against malaria in combination with standard malaria control
Source: Curr Res Parasitol Vector Borne Dis. 2025 Jan 29;7:100247. doi: 10.1016/j.crpvbd.2025.100247 (PMC11869851; doi:10.1016/j.crpvbd.2025.100247)
Supplement: Multimedia component 1 [file mmc1.pdf]

Supporting Information

Exploring the epidemiological impact of attractive targeted sugar bait in combination with standard malaria control:

Nima R. Moghaddas[a*], Mohamed M. Traore[b], Gunter C. Müller[b], Joseph Wagman[c], Javan Chanda[d], Julian Entwistle[e], Christen M. Fornadel[e], Thomas S. Churcher[a]

Supplementary Table S1. Parameters used in the deterministic ASB model and individual-based malaria transmission model. Mathematical notation for ITN parameters follows the conventions in Nash et al. (2021) and notation for IRS parameters follows the conventions in Sherrard Smith et al. (2018). Unless otherwise stated all other individual-based malaria transmission model parameters are taken from Churcher et al. (2024). Seasonality parameters were calculated from CHIRPS global rainfall rasters using the netz package (https://github.com/mrc-ide/netz). The effects of ${\mu_i}^{ATSB}$, f, r, ${\mu_i}^{dye}$, population usage of ITNs, coverage of IRS and pyrethroid resistance were explored in this study.

Intervention	Parameter definition	Median (range)	Reference
ATSB	Excess mortality due to ATSB ${\mu_i}^{ATSB}$	Varied	Explored in this study
ASB	Bait feeding rate per day f	Varied	Explored in this study
	Dye longevity r	4.5 days (2.1-7.0)	Explored in this study
	Dye mortality ${\mu_i}^{dye}$	0.0 per day (0.0-0.1)	Explored in this study
	Natural mortality ${\mu_i}^{nat}$	*Anopheles arabiensis*: 0.13<br>*Anopheles funestus*: 0.11<br>*Anopheles gambiae*: 0.13	White et al., 2011
Pyrethroid-only ITN	Killing rate d_{n0}	0.27 (0.18-0.32)	Churcher et al., 2024
	Maximum repellence r_{n0}	0.69 (0.65-0.74)	Churcher et al., 2024
Pyrethroid-pyrrole ITN	Killing rate d_{n0}	0.49 (0.18-0.54)	Churcher et al., 2024
	Maximum repellence r_{n0}	0.51 (0.45-0.63)	Churcher et al., 2024
All ITNs	Minimum repellence r_{nm}	0.24	Lines et al., 1987, Curtis et al., 1996
	Net efficacy decay γ_n	2.64 per year	Mahama et al., 2007
	Population usage following mass campaign	70% (50-90)	malariaatlas.org, explored in this study
	Mean net retention	2.5 years	Bertozzi-Villa et al., 2021
IRS (pirimiphos-methyl)	Maximum killing rate $k_{s\theta}$	2.03 (1.83 – 4.75)	Sherrard-Smith et al., 2018
	Killing rate decay $k_{s\gamma}$	-0.009 (-0.014 – -0.010)	Sherrard-Smith et al., 2018
	Maximum blood-feeding	-2.22 (-2.167 – -4.360)	Sherrard-Smith et al.,

	inhibition $l_{s\theta}$		2018
	Blood-feeding inhibition decay $l_{s\gamma}$	0.008 (0.006-0.014)	Sherrard-Smith et al., 2018
	Maximum repellence $k_{s\theta}$	-1.232 (-4.471 – -1.000)	Sherrard-Smith et al., 2018
	Repellence decay $k_{s\gamma}$	-0.009	Sherrard-Smith et al., 2018
	Coverage	40% (20-60)	Explored in this study
SMC (sulphadoxine-pyrimethamine and amodiaquine)	Probability of successfully clearing infection	0.90	Thompson et al., 2022
	Shape parameter of Weibull distribution	4.30	Thompson et al., 2022
	Scale parameter of Weibull distribution	38.10	Thompson et al., 2022
Site-specific parameters (Keyes province, Mali)	Seasonality	g_0: 2.36<br>g_1: -2.99<br>g_2: 0.16<br>g_3: 0.62<br>h_1: -2.38<br>h_2: 2.16<br>h_3: -0.27	https://www.chc.ucsb.edu/data/chirps
	Species composition	*An. arabiensis*: 0.45<br>*An. funestus*: 0.29<br>*An. gambiae*: 0.26	malariaatlas.org
	Proportion of cases successfully treated f_T	0.30	malariaatlas.org
	Pyrethroid resistance (survivorship in discriminatory dose bioassay)	0.70 (0.50-0.90)	malariaatlas.org, explored in this study
Site-specific parameters (Western province, Zambia)	Seasonality	g_0: 1.98<br>g_1: 3.13<br>g_2: 0.72<br>g_3: 0.29<br>h_1: 1.02<br>h_2: 0.77<br>h_3: -0.10	https://www.chc.ucsb.edu/data/chirps
	Species composition	*An. arabiensis*: 0.025<br>*An. funestus*: 0.95	Personal communication

		An. gambiae: 0.025	
	Proportion of cases successfully treated f_T	0.60	malariaatlas.org
	Pyrethroid resistance (survivorship in discriminatory dose bioassay)	*An. arabiensis*: 0.71<br>*An. funestus*: 0.80<br>*An. gambiae*: 0.71	Personal communication, explored in this study

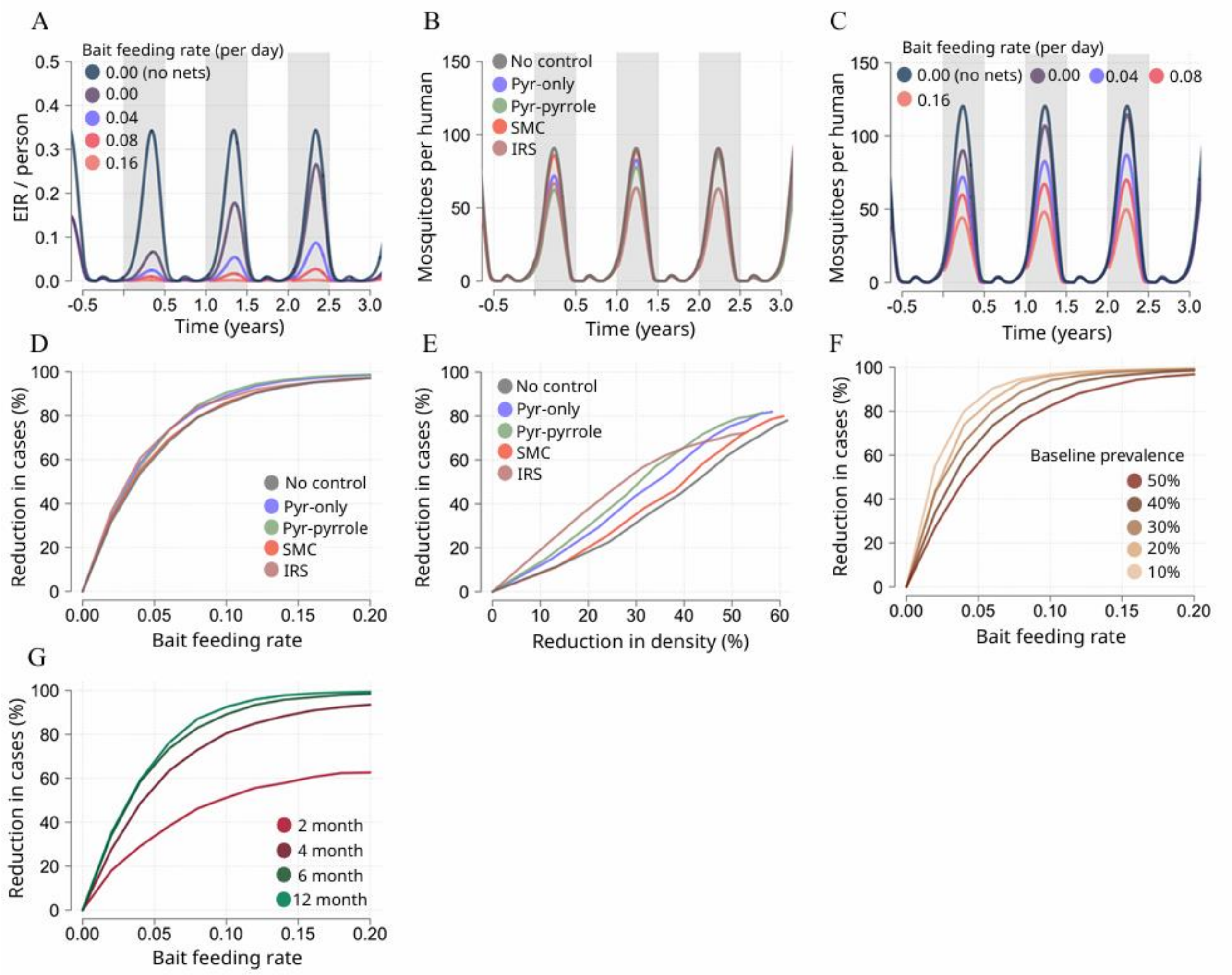

Supplementary Figure S1. Additional figures showing additional outputs for the scenario explored in Figure 1 of the main text. (A) Temporal dynamics for the entomological inoculation rate (EIR - infectious bites per person per day), over three years for different ATSB feeding rates. These are the same model simulations presented in Figure 2. (B-C) Mosquito density over time for deploying ATSBs on different interventions at a fixed feeding rate of 4% (B) and for different feeding rates with pyrethroid-only nets (C). Percentage reduction in all-age cases of clinical malaria relative to no ATSB as a function of feeding rate for different intervention scenarios (D) different baseline prevalences (F) and different lengths of ATSB deployment (G). Baseline prevalence was measured as the mean prevalence over the two months of highest transmission (F). ATSB deployment intervals were centered around the peak of the transmission season (G). Percentage reduction in clinical cases of malaria as a function of mosquito density for ATSB with different interventions (E).

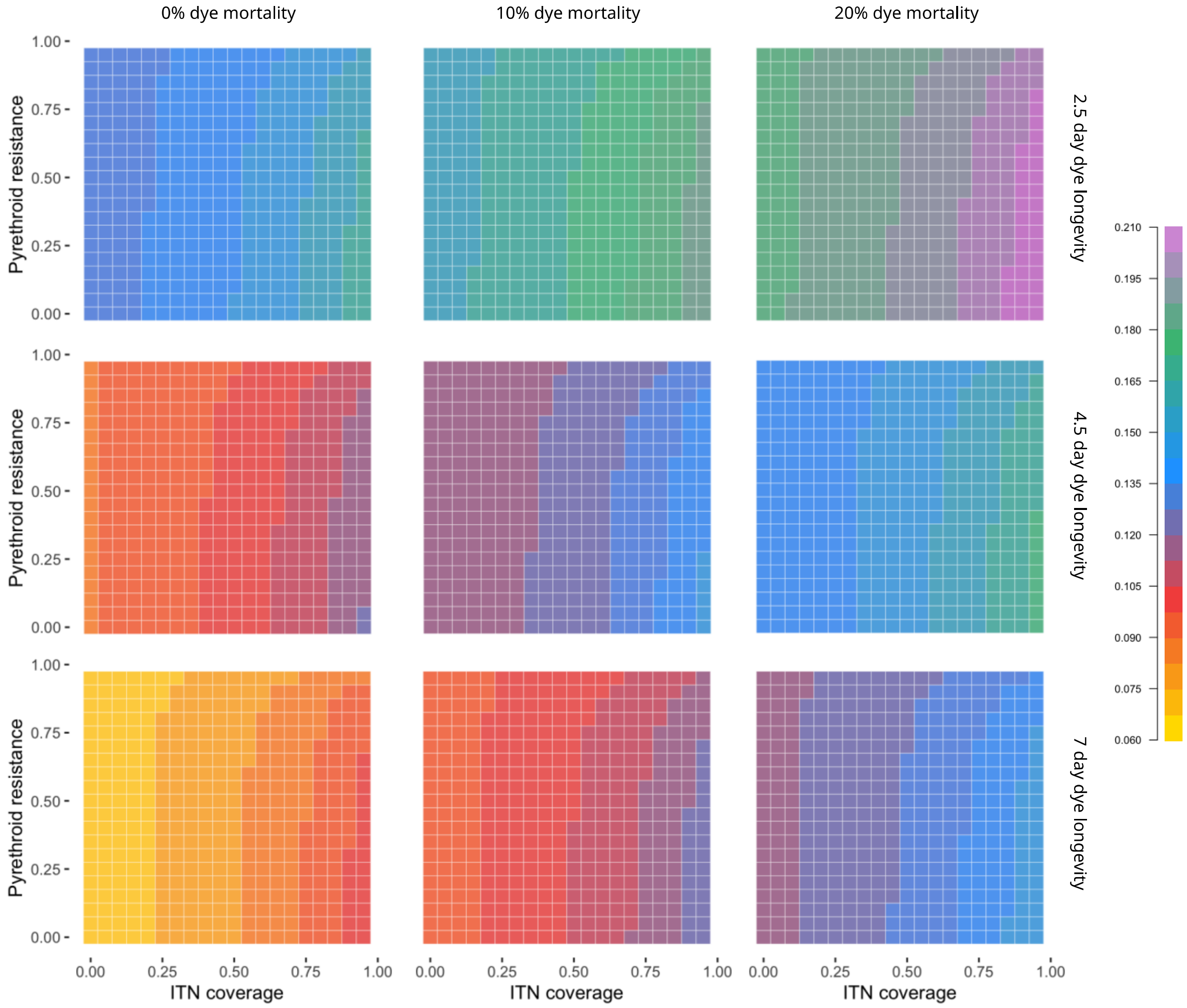

Supplementary Figure S2. Heatmaps of a sensitivity analysis showing daily bait feeding rates required to observe a 20% dyed fraction under a variety of assumptions on pyrethroid-only ITN population usage (referred to as coverage), levels of pyrethroid resistances (as described by survivorship in a discriminating dose bioassay), dye longevities and dye induced mortality.

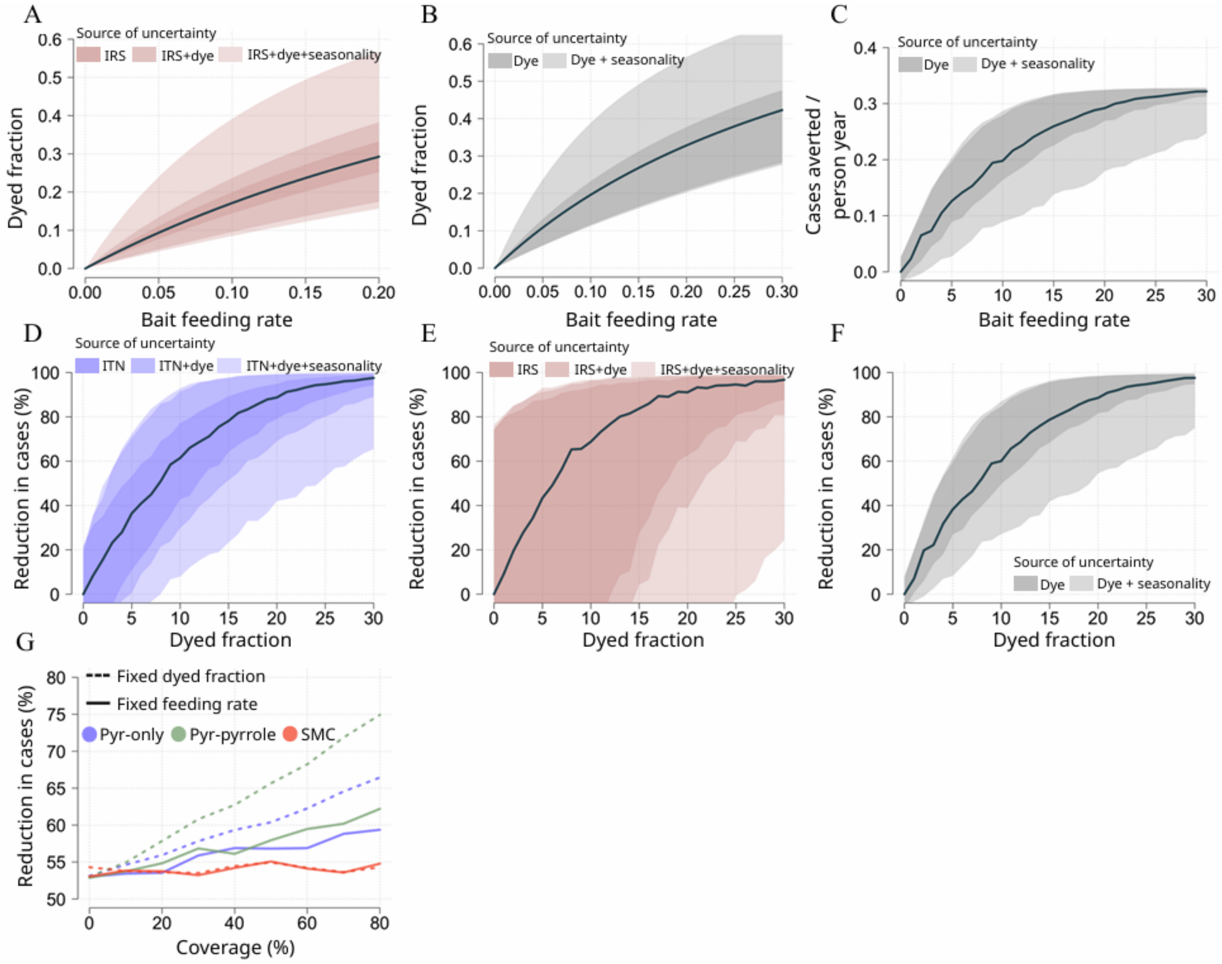

Supplementary Figure S3. Dyed fraction as a function of daily feeding rate with various sources of uncertainty arising from background IRS campaigns (A) and in the absence of alternative interventions (B). IRS uncertainty consists of variation in coverage (20-60%), dye uncertainty consists of variation in mean dye longevity (2.1-7 days) and dye mortality (0-10%), and seasonality uncertainty consists of variation in time of mosquito sampling (10 to 180 days post bait deployment). Percentage reduction in all-age clinical malaria cases as a function of dyed fraction for the same scenarios shown in Figure 3E of the main text (D), for the same scenarios shown in Figure 3F of the main text (E) and for the same scenarios as shown in panel C of this figure (F). Percentage reduction in all-age cases of clinical malaria relative to no ATSB as a function of ITN or SMC coverage for a fixed feeding rate (4%) or fixing the dyed fraction (10.7%) and allowing the feeding rate to vary with intervention coverage (H).

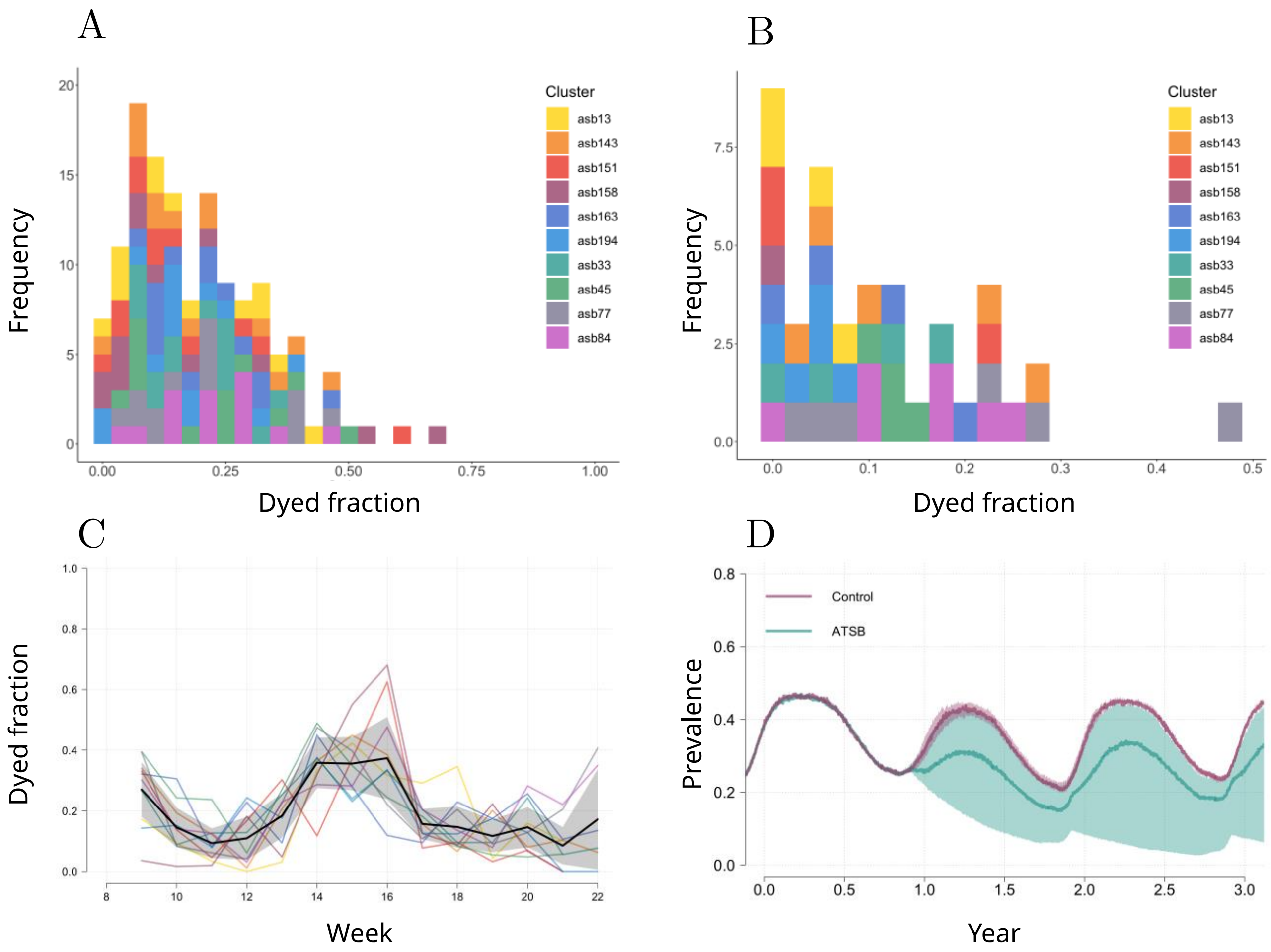

Supplementary Figure S4. Cluster level Zambian data and model predictions. Histogram of weekly dyed fractions observed across 10 different Zambian clusters for (A) *Anopheles funestus*, the dominant vector, and, (B) *Anopheles gambiae*. (C) Dyed fractions broken down by cluster and time for both species with the thick black line indicating the mean estimate for that time point. (D) Projected ATSB impact on prevalence of malaria in people aged 6 months – 25 years for a Zambia-like site already using pyrethroid-only ITNs with the full range of uncertainty shown in Figure 3B of the main text.

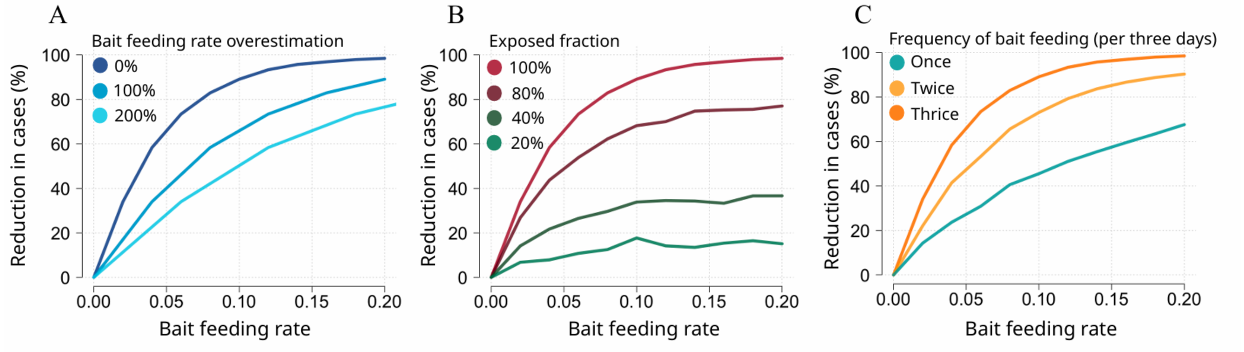

Supplementary Figure S5. Percentage reduction in all-age cases of clinical malaria as a function of feeding rate for the same scenarios as shown in Figure 5 of the main text. Panel (A) shows different levels of bait feeding rate overestimation, be it no bias (default model, dark blue line) or bias which could be caused by stained mosquitoes more likely to be caught than those transmitting malaria. Panel (B) shows varying proportions of the mosquito population which feed on bait stations, from 100% (no heterogeneity in mosquito population, default model, red line) to only 20% of the mosquito population feeding on bait stations (and caught in ASB traps) with the other 80% not feeding on bait stations (and not being caught in traps, green line). Panel (C) shows models which assume that the observed feeding rate happens every day (default model, orange line), twice every three days (yellow line) or once every three days (turquoise line).

References

1. Bertozzi-Villa, A., Bever, C.A., Koenker, H., Weiss, D.J., Vargas-Ruiz, C., Nandi, A.K., Gibson, H.S., Harris, J., Battle, K.E., Rumisha, S.F., Keddie, S., Amratia, P., Arambepola, R., Cameron, E., Chestnutt, E.G., Collins, E.L., Millar, J., Mishra, S., Rozier, J., Symons, T., Twohig, K.A., Hollingsworth, T.D., Gething, P.W., Bhatt, S., 2021. Maps and metrics of insecticide-treated net access, use, and nets-per-capita in Africa from 2000-2020. Nat Commun 12, 3589. https://doi.org/10.1038/s41467-021-23707-7
2. Churcher, T.S., Stopard, I.J., Hamlet, A., Dee, D.P., Sanou, A., Rowland, M., Guelbeogo, M.W., Emidi, B., Mosha, J.F., Challenger, J.D., Denz, A., Charles, G., Russell, E.L., Fitzjohn, R., Winskill, P., Fornadel, C., Mclean, T., Digre, P., Wagman, J., Mosha, F.W., Cook, J., Akogbéto, M., Djogbenou, L.S., Ranson, H., Manjurano, A., N'Fale, S., Protopopoff, N., Accrombessi, M., Ngufor, C., Foster, G.M., Sherrard-Smith, E., 2024. Projecting Epidemiological Benefit of Pyrethroid-Pyrrole Insecticide Treated Nets Against Malaria. https://doi.org/10.2139/ssrn.4569154
3. Curtis, C.F., Myamba, J., Wilkes, T.J., 1996. Comparison of different insecticides and fabrics for anti-mosquito bednets and curtains. Medical Vet Entomology 10, 1–11. https://doi.org/10.1111/j.1365-2915.1996.tb00075.x
4. Lines, J.D., Myamba, J., Curtis, C.F., 1987. Experimental hut trials of permethrin-impregnated mosquito nets and eave curtains against malaria vectors in Tanzania. Medical Vet Entomology 1, 37–51. https://doi.org/10.1111/j.1365-2915.1987.tb00321.x
5. Mahama, T., Desiree, E.J., Pierre, C., Fabrice, C., 2007. Effectiveness of Permanet in Côte d'Ivoire Rural Areas and Residual Activity on a Knockdown-Resistant Strain of Anopheles gambiae. Journal of Medical Entomology 44, 498–502. https://doi.org/10.1093/jmedent/44.3.498
6. Sherrard-Smith, E., Griffin, J.T., Winskill, P., Corbel, V., Pennetier, C., Djénontin, A., Moore, S., Richardson, J.H., Müller, P., Edi, C., Protopopoff, N., Oxborough, R., Agossa, F., N'Guessan, R., Rowland, M., Churcher, T.S., 2018. Systematic review of indoor residual spray efficacy and effectiveness against Plasmodium falciparum in Africa. Nat Commun 9, 4982. https://doi.org/10.1038/s41467-018-07357-w
7. Thompson, H.A., Hogan, A.B., Walker, P.G.T., Winskill, P., Zongo, I., Sagara, I., Tinto, H., Ouedraogo, J.-B., Dicko, A., Chandramohan, D., Greenwood, B., Cairns, M., Ghani, A.C., 2022. Seasonal use case for the RTS,S/AS01 malaria vaccine: a mathematical modelling study. The Lancet Global Health 10, e1782–e1792. https://doi.org/10.1016/S2214-109X(22)00416-8
8. White, M.T., Griffin, J.T., Churcher, T.S., Ferguson, N.M., Basáñez, M.-G., Ghani, A.C., 2011. Modelling the impact of vector control interventions on Anopheles gambiae population dynamics. Parasites Vectors 4, 153. https://doi.org/10.1186/1756-3305-4-153
